# The Impact of an Intergenerational Dance Project on Older Adults’ Social and Emotional Well-Being

**DOI:** 10.3389/fpsyg.2020.561126

**Published:** 2020-09-16

**Authors:** Louise Douse, Rachel Farrer, Imogen Aujla

**Affiliations:** School of Media and Performance, Research Institute for Media and Performance, University of Bedfordshire, Bedford, United Kingdom

**Keywords:** intergenerational, dance, social well-being, affect, basic needs

## Abstract

There has been strong interest in intergenerational arts practice in the United Kingdom since the 1980s; however, there is a generally weak evidence base for the effectiveness of intergenerational practice regardless of the domain. The aim of this study was to investigate the outcomes of an intergenerational arts project on participants’ social and psychological well-being using a mixed-methods, short-term longitudinal design. *Generations Dancing* brought together community artists with students (*n* = 25) and older adults (*n* = 11) living in Bedford. Over an 11-week period, participants worked together to produce a new dance performance and photography exhibition. Focus groups were conducted with the participants to explore their feelings about the collaboration across generations and communities. Participants also completed a battery of questionnaires preproject and postproject, to assess any change in their levels of well-being. Results indicate that the older adults showed increased confidence and willingness to connect with others; they got immense enjoyment from talking about their experience with others. Furthermore, the project helped to address negative stereotypes that the older adults had of working with the young people. The older adults enjoyed the students’ company and felt encouraged and supported by the young people. While a small number of challenges were identified, including difficulties in traveling to the workshops for vulnerable participants, most challenges were overcome through the older adults’ engagement in the project. For example, initial anxieties regarding the performance seemed insufficient to affect the participants’ overall enjoyment of the project. The findings were supported by the increased scores in relatedness, affect, and social well-being over time, but were not statistically significant. The results of this study indicate that intergenerational dance and arts projects can have wide-reaching positive impacts on both social and psychological well-being. However, there were a number of methodological challenges, including difficulty in recruiting sufficient numbers of both experimental and control groups for a robust quantitative evaluation of the data. These challenges highlight that “real life” settings and scenarios can influence the amount, nature, validity, and reliability of data collected. Going forward we encourage researchers to continue to consider innovative ways to address such methodological challenges.

## Introduction

The aim of this study was to investigate the outcomes of an intergenerational arts project on participants’ social and psychological well-being. This study is part of a larger project, *Generations Dancing*, which was facilitated by the University of Bedfordshire and brought together community artists with students from two local schools and older adults housed in assisted-living centers in Bedford. Over an 11-week period, participants worked together to produce a new dance performance and photography exhibition that was premiered at the University of Bedfordshire. The *Generations Dancing* project was aligned with the *Bedford Borough Health and Wellbeing Strategy* Bedford Borough Council (2014). The strategy outlines five priorities, four of which specifically address the health and well-being of young people (Priorities 1 and 2) and older adults (Priorities 3 and 5). As well as aiming to enhance participants’ psychological well-being, *Generations Dancing* sought to build on the council’s priorities by developing participants’ “sense of community, social capital, and civic safety” (Bedford Borough Council, 2014, p. 10). This article reports primarily on data regarding the older adults; data regarding the young people involved are reported in detail elsewhere (Douse et al., in preparation).

Previous research has shown the relationship between age and personal well-being to be U-shaped (ONS, 2016). That is, our sense of personal well-being is highest among younger people and older people and is lowest among people in their middle years. Despite this, it is important to remember that people 65 years or older represent a diverse group, with those older than 75 years particularly noting less satisfaction with health and personal well-being declining as people move into their 80s (ONS, 2018). Previous evidence has found that those 80 years or older were also twice as likely to report feelings of loneliness compared with younger age groups (ONS, 2015). In addition, these feelings of loneliness were found to have a strong relationship with low personal well-being ratings (ONS, 2015). Further research has reported a number of negative well-being outcomes associated with loneliness in older age (Bennett, 2002; Steptoe et al., 2004; Bolton, 2012; Duane et al., 2013; Hawkley et al., 2003). In addition to a declining sense of well-being, many older adults also face barriers to accessing the arts. The Department for Digital, Culture, Media and Sport’s Taking Part Survey (Department for Digital, Culture, Media and Sport, 2019) revealed that adults 75 years or older had engaged less than all other age groups, and a poll of older people 65 years or older commissioned by the Arts Council England (2016) revealed that well over a third found it more difficult to take part in arts and cultural activity now than when they were younger. Age UK (2018a) identified specific barriers that center around “location, transport, poor health (mental or physical), poor social networks, and low income.”

As a response to this, there has been strong interest in intergenerational practice in the United Kingdom since the 1980s (Beth Johnson Foundation, 2011), and in the early 21st century, a number of publications were produced by local government associations to support the development of such schemes and practices (Springate et al., 2008; Welsh Local Government Association, 2012; Hatton-Yeo and Melville, 2013; Clyde and Ryall, 2014; Manchester City Council, n.d.). Most of the publications refer to the Beth Johnson Foundation definition of intergenerational practice:

*Intergenerational practice aims to bring people together in purposeful, mutually beneficial activities which promote greater understanding and respect between generations and contribute to building more cohesive communities. Intergenerational practice is inclusive, building on the positive resources that the young and old have to offer each other and those around them.* (Beth Johnson Foundation, 2011, p. 4).

Research has reported a number of positive outcomes associated with intergenerational practice (Springate et al., 2008; Beth Johnson Foundation, 2011; Kagan et al., 2012; Welsh Local Government Association, 2012; Hatton-Yeo and Melville, 2013; Clyde and Ryall, 2014; Park, 2014; Dorsett et al., 2015; Whitehouse, 2017; Manchester City Council, n.d.). Key benefits include increases in health and well-being indicators, such as improved fitness and physical mobility among the elderly and increased confidence and self-esteem among younger participants (Robinson et al., 2006). There are also a number of outcomes cited around the development of community relationships, cohesion, and social capital, including a reduction in isolation and antisocial behavior and reduced fear of crime (Hatton-Yeo, 2008; YouthLink Scotland, 2011). In dance specifically, there have been a number of intergenerational projects in the last 1–15 years. A small number of publications have described such projects and detailed creative practices (Apol and Kambour, 1999; Borstel, 2006; Morelli, 2010). However, there is limited empirical research that specifically examines the impact of dance on participants’ well-being in intergenerational projects (Sherman, 1997; Schroeder et al., 2017). Indeed, there is a generally weak evidence base for the effectiveness of intergenerational practice regardless of the domain. For example, many studies are cross-sectional in nature, so causality cannot be assumed, have involved small sample sizes, and often did not recruit a control group. Springate et al. (2008, p. 18) in their systematic review of the literature state that “[t]here were few rigorous evaluations of projects in the United Kingdom, and there was a wide diversity in terms of what was evaluated and how the evaluations were carried out.” They recommend a more consistent framework for evaluations that allows for greater comparison across studies. The aims of the current study therefore are to evaluate the impact of intergenerational practice on participant well-being using a mixed-methods, short-term longitudinal design.

Numerous definitions of well-being exist in the context of positive psychology, with increasing distinction being made between hedonic well-being and eudaimonic well-being (Waterman, 1993, 2007; Ryan and Deci, 2001; Deci and Ryan, 2008; Ryan et al., 2008; Waterman et al., 2008; Henderson and Knight, 2012; Huta and Waterman, 2014). Hedonic well-being is often related to the experience of pleasure and happiness and uses the assessment of subjective well-being: the presence of a positive mood, the absence of negative mood and measures of life-satisfaction (Miao et al., 2013; Pavot and Diener, 2013). Eudaimonic well-being, on the other hand, is often considered to include hedonic well-being, in terms of the experience of happiness, but also includes self-realization, personal expressiveness, excellence, and relatedness (Huta and Waterman, 2014). The current study adopted measures to address both hedonic (positive and negative mood states) and eudaimonic (basic psychological needs satisfaction) aspects to reflect the multifaceted nature of well-being. In the context of older adult well-being, Age UK (2018b) completed a 5-year study (2009–2014) into what makes later life worth living. From this study, they produced the *Index of Wellbeing in Later Life* Age UK (2018b). One of the key findings from the research was “the importance of maintaining meaningful engagement with the world around you in later life” (Age UK, 2018b). Importantly, they found that creative and cultural participation was the most significant factor in contributing to older adults’ well-being. Dance appears to be particularly well-placed to provide meaningful creative and cultural participation and collaboration.

Many studies have shown that dance can play a significant role in improving the health and well-being of various populations including children, older adults (with and without preexisting medical conditions), and other recreational dancers, across North America, South America, Europe, and Asia and performing a range of styles including cultural dance, ballroom, contemporary, pop, and jazz (Olvera, 2008; Bupa, 2011; Burkhardt and Brennan, 2012; Arts Council England, 2014; Hwang and Braun, 2015; Crossick and Kaszynska, 2016; Karkou et al., 2017; Vella-Burrows et al., 2017). A number of studies have addressed both physical and psychological well-being among young people (Quin et al., 2007; Joynson et al., 2009; Keay and Spence, 2009; Nordin and Hardy, 2009; Blazy and Amstell, 2010; Connolly et al., 2011; Dance South West, 2011; Urmston et al., 2012) reporting improvements in physical, social, and psychological well-being, including increased flexibility, self-esteem, and intrinsic motivation (Connolly et al., 2011; Dance South West, 2011; Urmston et al., 2012). In older adults, similarly, research findings indicate that dance can result in improvements in fitness and a range of cognitive and social indices (Nordin and Hardy, 2009; Bupa, 2011; Vella-Burrows et al., 2017). There have also been a number of studies looking specifically at dance for older adults with a variety of pathologies (Nordin and Hardy, 2009; Bupa, 2011). Most notable is a series of publications by Sara Houston and Ashley McGill on the benefits of dance for people with Parkinson disease (Houston and McGill, 2012; Houston, 2014, 2015; McGill et al., 2014). These authors employed a mixed-methods approach including validated questionnaires, physical fitness testing, focus groups, and observations. Houston and McGill advocate for methodological eclecticism, given that outcomes can often be multifaceted, and a mixed-methods approach can offer “a richer picture of what is meaningful and important to participants” (Houston and McGill, 2012, p. 116). This suggests that a mixed-methods approach could be valuable in investigations of intergenerational practice, to generate a rich and comprehensive understanding of the benefits dance can have on participant well-being.

However, there is growing criticism of the absence of a tangible definition of well-being (Dodge et al., 2012) and recognition that well-being can be assessed in multiple domains, such as physical, emotional, social, and spiritual, but with much of the research focusing solely on emotional and psychological well-being (McDowell, 2010). While much of the research on intergenerational practice, older adult well-being, and dance and well-being articulates perceived social benefits, there is limited use of validated questionnaires to support this. There is also no agreed-upon definition of social well-being, and often multiple terms are defined and/or used interchangeably. For example, Levasseur et al. (2010) provide an analysis of 43 definitions of social participation, identifying taxonomy of six levels of participation. However, because of such limited agreement on definitions, few authors have produced measures of social well-being (Keyes, 1998; Fuller-Iglesias and Rajbhandari, 2016). Corey Keyes, who has published widely in the field of positive psychology (Ryff and Keyes, 1995; Keyes, 1998; Keyes et al., 2002; Keyes and Shapiro, 2004), has clearly defined social well-being and created a validated instrument of its measurement (1998). In his social well-being scale, Keyes proposes operational definitions and indicators of social well-being for the “appraisal of one’s circumstance and functioning in society” (Keyes, 1998, p. 122). Keyes argues that well-being is dependent on a number of social challenges; “therefore well-being includes social dimensions such as coherence, integration, actualization, contribution, and acceptance” (Keyes, 1998, p. 133). The questionnaire has been regularly cited and used with large sample sizes, demonstrating its validity and reliability. This article therefore aims to provide detailed data to address the impact intergenerational dance practice has on older adults’ psychological and social well-being, generated from a combination of quantitative and qualitative data.

## Materials and Methods

A short-term longitudinal, mixed-methods design was employed to better understand the social and emotional well-being of older adults. Mixed-methods designs provide greater breadth and depth of research, providing rigorous quantitative evidence (which is lacking in much intergenerational research), as well as capturing the context-specific and often complex qualitative data relevant to such projects (Jones et al., 2016). A one-phase triangulation design was adopted whereby qualitative and quantitative data were combined at the interpretation phase (Creswell and Clark, 2017).

### Procedure

The study was granted approval by the University of Bedfordshire Research Ethics Scrutiny panel. All participants were voluntary and gave written informed consent prior to taking part in the research, including parental consent for school-age participants. All participants were provided with information sheets prior to the start of the study, and participants were informed that they could withdraw from the research at any time and that they could abstain from answering any questions that made them feel uncomfortable.

The 11-week project consisted of two phases. Weeks 1–5 involved separate 90 min sessions once a week for both the older adults and the young people. The older adults were based at one of the independent living centers (ILCs) while the young people worked alternately across both schools. The sessions consisted of preparatory movement exercises, relationship building exercises, and the chance to make introductions between the older adults and young people via video recordings. Weeks 6–10 consisted of 90 min sessions once a week, which brought all participants together at the University of Bedfordshire. These sessions shaped the dance component of the performance and offered opportunities for those interested to take a more documentary approach by photographing the workshops and one another. The project culminated in a performance and exhibition in the 11th week showcasing the final intergenerational performance work alongside performances by other local adult dance groups and school groups, as well as an exhibition of the photography taken by both the photographer and by the participants. Sessions were led by two community artists, a dance artist familiar with working with older people and a local photographer who has worked on multiple interdisciplinary projects within dance. In addition to the two artists, sessions were supported by a number of student interns from the University of Bedfordshire.

In the initial recruitment phase for the older participants, four local care providers were contacted and two older adult activity providers. One local care provider responded (Bedford Sheltered Housing Association) inviting us to work with three of their local ILCs, and one older adult activity provider responded (UoB Next Chapter Dance Group). Older adult participants were recruited using information sheets designed by the lead investigators and delivered by both lead investigators and gatekeepers. The lead investigators attended several coffee mornings at the ILCs and one class at the UoB Next Chapter Dance Group to provide further information for the older adults and to gather information on any access needs or requirements prior to the project start. Interested participants (*n* = 12) returned a signed consent form and were then contacted directly by the lead investigators. Control participants (*n* = 6) were recruited from the same groups as the experimental participants. Control participants gave consent to complete the questionnaire data but did not participate in the project. Inclusion criteria were as follows: participant is willing and able to give informed consent for participation in the study; male or female, 60 years or older; and living locally to Bedford. During phase 2 of the study, three of the participants from the experimental group (25%) and three participants from the control group (50%) dropped out.

For the young people, two local schools were contacted as part of the University of Bedfordshire’s National Collaborative Outreach Programme, and both responded. The young people (*n* = 23) were recruited using information sheets designed by the lead investigators and delivered by the gatekeepers. Interested participants returned a signed consent form. Control participants (*n* = 13) were recruited from the same groups as the experimental participants. Control participants gave consent to complete the questionnaire data but did not participate in the project. Inclusion criteria were as follows: parent/legal guardian is willing and able to give informed consent for the young persons’ participation in the study; male or female, school-aged; living locally to Bedford. During phase 2 of the study, four of the participants from the experimental group (17%) and one participant from the control group (8%) dropped out.

### Qualitative Methods

Focus groups were conducted separately with seven older adults, three scheme leaders, eight young people, two teachers, and two community artists. The older adults were recruited from three ILCs and a university-run older-adult dance class. The three scheme leaders were recruited from the three ILC’s. Both the students and teachers were recruited from two local schools. One of the community artists was local to Bedford, and the other from the North of England. Focus groups were arranged at times and places convenient to the participants.

A semistructured interview guide was created for each group of participants (see Supplementary Appendix 1), divided into three sections: introductory questions and background information on previous experience of similar projects; feelings about the collaboration across generations and communities; including questions about their experiences of the project, what they found positive and negative about it, their experience of collaborating with others their own age and with those of a different generation, their understanding of citizenship and community within the scope of the project; and the role of the university in the organization and management of the project (which is reported on in Farrer et al., under review).

Focus group interviews lasted between 20:22 and 1:12:27 min and were transcribed verbatim and uploaded into NVivo software (QSR, Melbourne, Australia) for inductive analysis. Transcripts were read thoroughly, and all relevant segments of text were coded into meaningful units, which were then placed into logical categories (themes). Lower-order themes were combined to form higher-order themes, and this hierarchy was constantly updated and refined as part of the analysis process (Patton, 2002). All of the transcripts were coded by the first author, and the second author independently coded 15% of the transcripts to ensure parity and agreement between the researchers (Creswell and Miller, 2000).

### Quantitative Methods

Participants completed a battery of questionnaires preproject and postproject, to assess any change in their levels of well-being. Descriptive demographic data for each group that completed questionnaires can be found in Table 1.

**TABLE 1:** Demographics of the sample.

	**Older adults experimental (*n* = 12)**	**Older adults control (*n* = 6)**	**Young people experimental (*n* = 23)**	**Young people control (n = 13)**
Age (years)	81.75 ± 11.48	85 ± 5.59	13.09 ± 0.79	14.15 ± 1.21
Ethnicity (n)	11 White	6 White	21 White	12 White
	1 Asian or Asian British		2 Black or Black British	1 Mixed race
			1 Asian or Asian British	
			1 Other ethnic group not specified	
Previous dance experience (%)	None: 50	None: 66.7	None: 4	None: 23.1
	<6 months: 0	< 6 months: 0	< 6 months: 8	< 6 months: 30.8
	6 months to 2 years: 16.7	6 months to 2 years: 0	6 months to 2 years: 4	6 months to 2 years: 7.7
	> 2 years: 33.3	> 2 years: 33.3	> 2 years: 84	> 2 years: 38.5
Currently dancing (n)	3	0	21	6
Hours per week in dance	1	0	1.90 ± 0.89	1

#### Materials

##### Demographic data

Participants provided information on their age, ethnicity, whether they had any previous dance experience, whether they were currently participating in any dance activity, and if so which dance style it was and how many hours per week they participated.

##### Basic needs

The Basic Psychological Needs Satisfaction Scale (Deci and Ryan, 2000) is a 21-item scale consisting of three subscales capturing data on participants’ feelings of autonomy, competence, and relatedness. It is scored on a 7-point scale from 1 (not at all true) to 7 (very true). Validity and reliability of the scale is published elsewhere (Deci and Ryan, 2000; Gagné, 2003). In the current study, at Time 1, the autonomy and competence subscales exhibited good internal reliability (α = 0.82 and 0.78, respectively), whereas an α level of.66 was accepted for the relatedness subscale as item deletion did not improve reliability. At Time 2, Cronbach α was satisfactory for autonomy (α = 0.75) and competence (α = 0.70 on deletion of one item) and acceptable for relatedness where again item deletion did not improve reliability (α = 0.69).

##### Affect

Participants’ affect was measured using the Positive and Negative Affect Scale (PANAS; Watson et al., 1988). The scale consists of two 10-item subscales tapping positive and negative affect, which are scored on a Likert scale from 1 (very slightly or not at all) to 5 (extremely). Participants were asked to complete the scale in reference to how they felt right now. The scale exhibited good internal reliability at both Time 1 (α = 0.78 for positive affect and α = 0.87 for negative affect) and Time 2 (α = 0.85 for positive affect and α = 0.90 for negative affect). The PANAS has published reliability information (Watson et al., 1988).

##### Social well-being

Social well-being was measured using the short (15 item) version of the Social Well-Being Scale (Keyes, 1998). The scale consists of five subscales, each with three items: social integration, social acceptance, social contribution, social actualization, and social coherence, scores from which were summed to create an overall score for social well-being. Items are scored on a 6-point Likert scale from 1 (agree strongly) to 6 (disagree strongly). Validity and reliability information is published in Keyes (1998). At both time points, scale reliability was good (α = 0.78 at Time 1 and α = 0.80 at Time 2).

#### Procedure

The scales were initially piloted with an independent group of older adults and young school-aged people to ensure that they were appropriate and that items were easily comprehended by each group. As a result of the pilot, some definitions were added to words for clarification, and the font size was increased so that the text was easier to read for the older adults. The questionnaires were completed at a time and location convenient to each group, at Time 1 (1 week prior to project commencement) and at Time 2 (1 week after the performance). Some older adults required help completing the questionnaires because of sight difficulties; the researchers or student interns assisted in these cases. Control groups were also recruited for both age groups in order to provide a comparison group and increase the robustness of the findings. However, these groups were difficult to recruit, and the resulting N is small.

#### Analysis

Data were first cleaned and screened for errors and outliers using IBM SPSS version 22. Although initially a multivariate analysis of variance was planned to compare the groups over time on the well-being measures, the sample size was insufficient to meet normality assumptions. Paired-samples *t-*tests were instead conducted to assess any differences in scores over time for the young people; a Wilcoxon rank test was used for the older experimental adults due to the small sample size (analyses of change over time for the older control group was not possible due to insufficient sample size). Independent-samples *t*-tests were then run to compare any differences in mean scores between the experimental and control groups of young people (comparisons between the older adult experimental and control groups were not possible due to the small N for the control group). A Bonferroni-adjusted α of 0.008 (0.05/6) was applied to mitigate the risk of type I error.

## Results

### Qualitative Results

**FIGURE 1:**
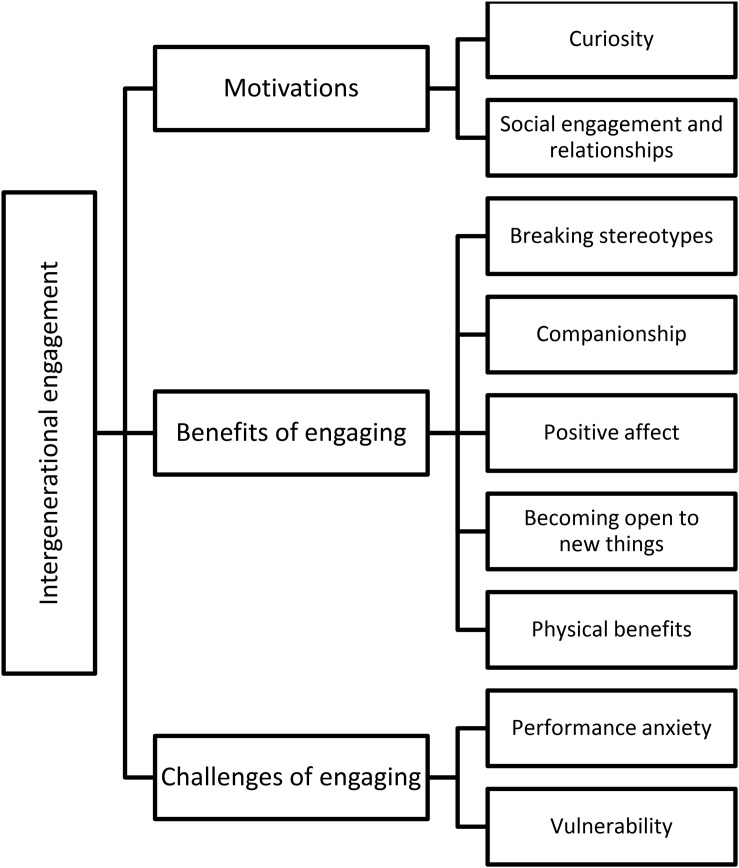
Model of intergenerational engagement.

The focus groups yielded a range of findings that were organized into three higher order themes: motivations, benefits of engaging, and challenges of engaging. Each theme is now reported in detail (see Figure 1).

#### Motivations

This theme was created from lower-order themes relating to the participants’ initial perceptions of and motivations for engaging in the project. Specifically, the older adults articulated positive motivations including a general curiosity or interest in the project and a desire for increased social engagement and relationships.

##### Curiosity

The older adults’ interest in the project was expressed in initial meetings and through the focus groups. Respondents cited being generally curious about the project and being encouraged by other members of the care home, or relatives:

*Well it was really curiosity. We didn’t know anything about it.* (Older adult)

Other participants commented on their interest in learning new skills, including the opportunity to engage in dance and to learn about new technologies; “I wanted a little more about the cameras. How to use them, the film, and take photographs. How to use*—*what do you call those?… Tablets” (older adult).

##### Social engagement and relationships

The older adults often reflected on their decreasing engagement with the community, articulating their current fears or realities about feeling lonely and disconnected from society. Despite regular organized entertainment every couple of months at the two ILCs, and coffee mornings and social afternoons, the participants commented that the communal areas were rarely occupied by the other ILC residents and that they could go for days without seeing anyone.

*I’m quite happy to be by myself. But it’s so [pause] when you come walk down here, there’s not a soul about. Saturday and Sunday—very, very quiet. There’s nobody sitting here.* (Older adult)

*You get a bit lonely, you see, on your own and all that.* (Older adult)

This therefore made it difficult for the older adults to make meaningful relationships with others: *“Because it’s hard, you know, to make friends”* (Older adult). Others attributed their disconnection with the broader community to a prior lack of interest or opportunity, or perhaps fear of engagement:

*Well I’ve never been one for doing things, like, with others.* (Older adult)

… *you know I’d always lived in my house, so [pause] and I’ve never mixed very much.* (Older adult)

This was reflected in the artists focus group, where they articulated how their understanding of community and citizenship had informed the work:

*I think in today’s world, all of us feel very disconnected to our community because, if you reflect back on history, everybody knew their neighbors; everybody knew their local shopkeepers, um, there wasn’t much moving around, around the country, where today, because people tend to move to different cities away from the family’s homes, um, we don’t tend to talk to our neighbors because we’re kind of all scared. I think there is a lack of feeling of belonging to an area*… *And I think because people are more scared, because there’s a lot more awareness of what goes on, where back in the day you wouldn’t know necessarily all the bad stuff going on, I think people are tending to keep themselves to themselves. So I think this project was an ideal situation to bring two groups together who would be scared themselves.* (Artist)

Some of the older adults were new to the area, or new to the ILC, and the scheme leaders commented that their attitudes toward leaving the ILC had changed; for example, over time the older adults became more reluctant to leave to go to the shops, and even attendance at previously organized events by the scheme leaders had been described as “hit and miss” (scheme leader). Therefore, the older adults’ attitudes and engagement with the broader community had seemingly waned; their awareness of this lack of engagement had been a motivating factor. As one participant explained:

*I like being with older people and children, so it seemed like a good combination.* (Older adult)

#### Benefits

##### Breaking stereotypes

One of the aims of the project was to address negative stereotypes that the older adults may have had of the young people and vice versa in order to enhance social integration and acceptance. In the initial 5 weeks where the artists worked separately with the older adults and young people, a series of activities were used to help the participants understand one another before the groups came together. The artists had asked each group of participants what advice they would give to the other group, and this was a very revealing exercise that highlighted some of the stereotypes they each held:

*Because, you know, even the older adults, at one point, when we were talking about, um, what is the stereotype of young people, what would you advise young people? They really were saying, “Oh young people aren’t connected, they’ve got fake friends.” And then when we told the young people this, they were so shocked and were like, “Well I disagree, because I meet up with my friends all the time, I have real good friends, we love hanging out together.” So, it’s just breaking that barrier.* (Artist)

Through the use of the camera work, opportunities were developed for each group to share something about themselves prior to their first combined session. However, there had been some nervousness prior to their coming together.

…*there was a lot of nervousness around that on both sides. Um, and even [pause] even that, when they [pause] you know, the two groups first came together, you could sense that nervousness, but actually they worked so well together.* (Artist)

During the focus groups with the older adults, there was an acknowledgement that their expectations had been challenged and most importantly that they had really enjoyed being with the young people.

*The young children were so good. You know, I thought they’d be a bit more airy, being with older people, um, but they were all extremely welcoming and they helped us, encouraged us*… (Older adult)

*Um, it was a long time since I’ve worked with children, although I spent my whole career doing that. And I thought that was just so charming, because they [pause] um, they were so unaffected. They were in no way, sort of, um, you know treading warily around. They were going “come on, take your hands,” whatever, and I thought that was just lovely. Their participation was brilliant.* (Older adult)

The younger people also commented on their enjoyment of working with the older adults and how it had enabled them to develop a shared understanding of one another:

*And it was, like, nice to interact with the older adults, as well, because, like, you never really see them.* (Young person)

*And I think, like, this kind of sounds a bit weird, but I think it helps us, like, understand, like, each generation more, because, like, I never met them, so I don’t really know their thoughts, but they [pause] because they’re, like, different generation, so they might think that our views are maybe, like, different and, like, wrong [pause] not wrong, but like weird and different to them, and then their views, like, might be, like, different to us. So it’s a good, like, understanding point.* (Young person)

This was also perceived by the artists working with them:

*And then when you think it can’t get any better, and you bring them together, again that whole shyness happens, and then by the end of the session they’re all chatting, they’re all helping the older adults out, and the older adults are smiling, and [pause] I just think it’s been a great reminder for myself of the power of the process. The power of dance. And then also just [pause], it just shows the power of, like, mixing communities and breaking stereotypes.* (Artist)

##### Companionship

In addition to enjoying spending time with the young people, many of the older adults commented on how much they enjoyed getting to know people of a similar age and being involved in doing something with others:

*Most of [pause] most of it was the company. Being with other people, doing what other people were doing. Being part and parcel of it all.* (Older adult)

*Yeah, it was nice really, wasn’t it? All the people who are sitting here, we know so well. We all know each other. We all get on. Nothing else [pause] that’s the start and finish of it.* (Older adult)

Some also commented on how it had also made an impact on how they engaged with others outside of the project:

*And so now, I’m beginning to mix with people, you know, “Good morning, how are you, are you off out today?” Things like that.* (Older adult)

This demonstrates an overall increase in confidence and willingness to engage with others which is supported by data from the following theme.

##### Positive affect

The older adults’ enjoyment of the project was expressed throughout the interviews and was evident not only in their descriptions of the project itself but also in their enthusiasm to talk about their experiences with family, friends, and the scheme leaders and carers:

*Um, they’ve all looked forward to it. They tell me every time they’ve been, what they’ve been doing. I have the rundown of it.* (Scheme leader)

*I think it gave people something to think about. It gave them another aspect, didn’t it, in their discussions and the talking. And you’d hear this, actually, at lunches*… *because people would then be discussing about what was going to be happening, and if they were coming down. So, yeah, no, it became a focal point.* (Scheme leader)

There were frequent mentions of positive outcomes including feelings of enjoyment and excitement as well as being inspired and encouraged. The older adults also referred to feeling more confident and proud of their achievements:

*I’m on a high when I go back. I don’t know what it does, but it does something.* (Older adult)

*I loved it. I loved it. It was a real, um, challenge and a real experience, you know. Like I said in the beginning, I never thought we would ever come close to doing anything like this.* (Older adult)

*Well, we felt that we was*… *proud, and in heaven, sort of thing, doing it.* (Older adult)

This included their enjoyment of the final performance:

*Oh, well, the performance. Oh yes, I think it was wonderful. It really was. You put such great effort into it, helping us and doing for us. It was beautiful, it was. I mean everybody cheered, didn’t they? Everybody*…

The artists had also observed increased confidence in the older adults:

*They’ve had, like, so many milestones along the way that there were some people*… *sitting at the back, but they were part [pause] they slowly kind of would involve them [pause] grew in confidence, that they would kind of stand up and walk in a little bit, you know, and kind of start to engage and interact and push themselves even, you know - whatever that is for them.* (Artist)

The scheme leaders observed positive responses from the participants, noting that they had not received any negative feedback:

*I think everybody that went, without a doubt, I felt enjoyed it.* (Scheme leader)

*The ones that took part, whether it was right to the end or not, nobody criticized [pause] you know, it was all positive feedback. They enjoyed it.* (Scheme leader)

##### Open to new things

Many of the participants described the pleasure they got from doing something new or out of their comfort zone:

*And it got you doing different things.*… *And, also to do something completely different from the activities that are provided here.* (Older adult)

*There’s no pressure. So I think that’s great. And it’s opened my eyes.* (Older adult)

This was reiterated by the scheme leaders, who were surprised at some of the older adults’ engagement. They commented on how one particular participant was not planning on doing the project, and they were doubtful if the participant would ever engage in such activities outside of the ILC, but after joining in, they commented on how much the participant enjoyed it. This also led the scheme leaders to discussions of classes they might run in the future:

*This activity is obviously not something they would ever have thought of, um, which is kind of open [pause] opened it up, so they may well be more receptive to anything else that comes along similar, or that involves things in the community a bit more.* (Scheme leader)

##### Physical benefits

The physical benefits of dance for older adults are well documented in the literature (e.g., Hwang and Braun, 2015), and as such was not a particular focus of this research project. However, anecdotally, participants expressed joy in feeling greater vigor and flexibility:

*And sometimes I’m tired, but I feel so invigorated when we finish.* (Older adult)

*Well I feel a bit more supple, I did. Because I’d got very stiff. And that helped.* (Older adult)

#### Challenges

The participants also articulated some of the challenges they faced during the project including some apprehension about the performance.

##### Performance anxiety

One of the biggest challenges to engaging in the project and initial anxieties of participants was around the performance at the end of the project:

*I’ve never performed. So this was a big challenge. I was very, um [pause] I wasn’t very keen on doing the actual performance.* (Older adult)

*And I remember like, in week 1, I think it was, one of the older adults was like, “Oh there’s going to be a performance, I don’t know if I’m going to [pause] I don’t know if I’m going to do that.”* (Artist)

Some of their anxiety arose from feelings of inadequacy in terms of the dance practice, “There were so many times I was not going to do it, because you think of dance and performance as showing off and more professional” (older adult). This quote suggests that the older adults had certain perceptions of dance and professional dance performance, which would typically have excluded them from participating. Participants also commented that their initial expectations were of a smaller-scale project and more like a weekly Keep Fit class: “I didn’t expect it to be on the scale that it was. I thought it would just be [in the ILC]” (older adult).

##### Vulnerability

One of the key challenges cited by the older adults was their difficulty when leaving the ILC to join the sessions on the university campus. As a result, three of the older adults ceased engagement. One of the key reasons cited was difficulty with walking the distance between the taxi drop-off and the dance studio:

*But we had the taxi, didn’t we, which was very nice. But it was too far away from the hall, and that’s what I find difficult. To walk. And then when I got there, I was alright [pause] lovely to come in, walked, dancing, music—yes, I loved that. But it was standing out. And when I got there, it was a very hot day.* (Older adult)

This was acknowledged by both the artists and scheme leaders, who were concerned for some of the more vulnerable older adults; “The constraint for the older adults—feeling confident enough to come out of the care home, which I didn’t expect…” (artist). The scheme leaders also acknowledged concerns from one of the older adults’ family, which prevented them from taking part outside of the ILC: “…but the family are always very, very frightened for her to come out of that environment without them being there […] Um, and it was that vulnerability…” (scheme leaders). However, the scheme leaders still felt that whereas some of the older adults stopped coming, they still benefited from engaging:

*and the fact that they really liked it when they weren’t doing it here, so okay, they’d stopped a bit prematurely, for them, that was their choice, but they don’t [pause] you know, they still got a lot out of it.* (Scheme leader)

### Quantitative Results

The means and standard deviations for each questionnaire variable and group are reported in Table 2, along with the N for each variable. The variability in attendance over the course of the project is reflected in the number of responses to each scale, and missing data suggest that the young people in particular did not engage well with the Basic Psychological Needs Satisfaction (BNS) scale. It may also be possible that, despite successful piloting of the scales, there were some difficulties in comprehension of some items for both groups, which participants may not have wished to share with the research team. As a result, the statistical analyses were conducted on small sample sizes (particularly the control groups, whereby there were insufficient cases of older control adults to conduct analyses). Taken together, this means that reported findings should be interpreted with caution. These factors are indicative of the challenges in collecting quantitative data with multiple community groups whose attendance and engagement may fluctuate. Perhaps unsurprisingly, therefore, there were no significant differences over time according to the paired *t*-tests and Wilcoxon rank tests or significant differences between the groups according to the independent *t*-tests (full results of the statistical analyses are shown in Supplementary Appendix 2).

**TABLE 2:** Mean scores for each group.

	**Autonomy**		**Competence**		**Relatedness**		**Positive affect**		**Negative affect**		**Social well-being**	
	**Time 1**	**Time 2**	**Time 1**	**Time 2**	**Time 1**	**Time 2**	**Time 1**	**Time 2**	**Time 1**	**Time 2**	**Time 1**	**Time 2**
Older adults experimental (*n* = 4–11)	5.171.09	6.070.78	4.401.30	4.870.96	5.880.78	6.600.61	36.185.96	38.636.35	13.644.06	12.755.01	58.739.43	63.6713.13
Older adults control (*n* = 0–4)	6.390.44	6.340.20	4.752.47	4.8	5.080.44	No data	29.4011.78	39.506.36	10.400.89	15.001.41	49.504.43	57.00
Young people experimental (*n* = 9–23)	4.821.17	4.651.31	4.731.51	4.741.14	6.040.59	6.070.55	33.986.94	31.007.01	17.578.10	16.367.55	58.0812.37	58.3110.71
Young people control (*n* = 4–13)	4.671.19	5.370.26	5.040.89	5.520.50	5.691.02	5.840.64	31.194.95	29.456.38	15.853.26	14.413.98	62.6712.16	62.5410.53

However, although there were no significant results, inspection of the means indicates that there were increases in every well-being variable (and a decrease in negative affect) for the older adult experimental group, including an increase of just over four points in social well-being. This is in accordance with the qualitative data; it is possible that these increases may have been significant with a larger sample size.

## Discussion

The aim of this study was to investigate the outcomes of an intergenerational dance and photography project on participants’ social and psychological well-being. Overall, analyses revealed that participants’ experiences of *Generations Dancing* were very positive with praise for the supportiveness of the people involved, the final event, and the opportunity for connection that it provided. The few challenges that were identified centered around the vulnerability of particular individuals, who found it difficult to leave the ILCs and some who expressed initial anxiety toward the final performance but for whom every single participant positively commented on after the event. The project emphasized collaboration and the breaking down of barriers and stereotypes across generations, with a view to developing participants connection with Bedford and the local community.

### Social Integration

One of the key outcomes of *Generations Dancing* has been the increased integration of the older adults within the ILCs in order to make meaningful contributions within their own social networks. As a component of social well-being, Keyes defines social integration as “the quality of one’s relationship to society and community” (Keyes, 1998, p. 122). He argues that healthy individuals feel that they belong to a community and are part of a shared social reality. This is evident in the older adults’ increased confidence and willingness to connect with others outside of the project, including their increased daily communication with other older adults, with scheme leaders and carers, and with friends and family. It seemed that the older adults had immense enjoyment from talking about their experience to others, and the project became a focal point for further discussions. This supports existing literature that intergenerational and arts activities are beneficial for improving social networks (Kagan et al., 2012; Clyde and Ryall, 2014; Park, 2014). Conversely, Keyes argues that social isolation and estrangement are the antithesis of social integration, and it is crucial to note that some of the participants acknowledged feelings of loneliness and a desire to develop more connections as key motivations for taking part in the project. It is evident that projects such as *Generations Dancing* can play an important role in providing social engagement and shared experience. The fact that some participants themselves listed this as a motivating factor indicates a real need for increased social activity among this population.

### Social Acceptance

Through intergenerational interaction and mutual learning, this project also offered opportunity for participants’ perspectives of others to be shifted and assumptions to be challenged. Keyes (1998) defines social acceptance as the generalized character or quality an individual may hold of other people: “Individuals who illustrate social acceptance trust others, think that others are capable of kindness, and believe that people can be industrious” (Keyes, 1998, p. 122). While many of the participants began the project with certain preconceptions, it was clear that the stereotyping of young people by the older adults was easily overcome through the use of preparatory workshop exercises. This is supported in the literature, which articulates the importance of preparatory exercises to enable intergenerational connection and understanding (Springate et al., 2008). Another key component of social well-being that was particularly evident in this project is that of social acceptance. The older adults also enjoyed the students’ company and sharing stories with them. They felt encouraged and supported by the young people. This benefit of intergenerational practice is widely reported in the literature (Springate et al., 2008; Welsh Local Government Association, 2012; Hatton-Yeo and Melville, 2013; Clyde and Ryall, 2014) and supports Age UK’s report that creative and cultural participation as a means for engaging with others is a significant contributor to well-being. It is also supported by the increased scores in relatedness and social well-being over time, despite not being statistically significant.

### Positive Emotions

Participants reported numerous positive emotions, including enjoyment excitement, confidence, and pride. These related to various experiences including the success of the final performance and the joy of seeing everyone applauding, as well as the positive interactions they had with others, such as their fellow peers, the young people and the supporting artists and volunteers. This is supported by the PANAS scores, which indicated a trend toward increases in positive affect and decreases in negative affect. This supports existing literature that intergenerational and arts activities are beneficial for the psychological well-being of individuals (Springate et al., 2008; Welsh Local Government Association, 2012; Hatton-Yeo and Melville, 2013). The research undertaken as part of *Generations Dancing* uniquely demonstrates this within the context of dance by evidencing the positive impact of dance on participants’ well-being. Furthermore, it became evident that the adults’ engagement in the project allowed them to break limiting beliefs and to become open to new things. Learning new things is associated with psychological well-being in terms of growth, accomplishment, and achievement (e.g., Ryff and Keyes, 1995; Seligman, 2011). This is further supported by the improved competence scores over time.

### Challenges

The challenges reported by the participants included anxiety toward the performance. However, the older adults also expressed enjoyment and feelings of pride after the event. Performance anxiety is common, even among professional dancers (e.g., Walker and Nordin-Bates, 2010), and demonstrates how much the older adults cared about doing a good job and how much the project meant to them. Other challenges included obstacles to participation with regard to access and safeguarding in terms of transport issues, location of classes, and communication with other family members; this is reported on further in Farrer et al. (in preparation).

### Limitations

The results of this study indicate that intergenerational dance and arts projects can have wide-reaching positive impacts on both social and psychological well-being. However, there were a number of methodological challenges, including difficulty in recruiting sufficient numbers of both experimental and control groups for a robust quantitative evaluation of the data. The questionnaires themselves were also challenging for some of the older adults, which was not evident in the initial piloting. For example, participants often struggled to complete the PANAS scale, not knowing how to interpret the Likert scale, but did always ask for help. Some of the phrasing of both the PANAS items and social well-being items was problematic for the older participants, with some interpreting them as personal attacks; i.e., when rating their feelings of guilt, participants would react with anger about what they perceived they were being accused of. As such, the validity of the responses from some participants is questionable. These various methodological challenges highlight that, despite our attempts to establish robust quantitative data, “real life” settings and scenarios can influence the amount, nature, validity, and reliability of data collected. In order for intergenerational projects to provide a meaningful and viable opportunity for participants, they typically need to involve short time scales and small numbers, which limit the amount and types of data that can be collected. Going forward, we encourage researchers to continue to consider innovative ways to address such methodological challenges.

However, in terms of the findings of this article, the qualitative and quantitative data did broadly support one another. It also important to note that the experimental older adults already had relatively high levels of well-being indices at the start of the project, reporting scores that were higher than those in previous studies of older adults in terms of basic need satisfaction (Goulimaris et al., 2014), positive affect (Mather and Knight, 2005; Parker et al., 2008), and social well-being (Keyes, 1998). This may in part explain the lack of significance in change over time. This also raises a final methodological question regarding potential differences between participants who self-select for projects like these and those who are less likely to put themselves forward, but may actually benefit more from the project. Recruiting “hard to reach” participants continues to be a challenge in such contexts.

## Conclusion

The impact of the *Generations Dancing* project has been overall very positive, drawing from a range of quantitative and qualitative information to paint an interwoven web of understanding and experience from the older adults’, young people’s, artists’, and teachers’ perspective. What is clear is the social value of a project like this being placed in locations where there is little existing provision for positive intergenerational interaction. Interview data from the older adults would suggest that this social value is far reaching, as they describe their growing number of interactions with peers, young people, friends, and family beyond the actual project sessions themselves. This research highlights the role that arts can play in connecting communities and encouraging social integration. It acts as a medium for engaging participants, but also a stimulus that encourages communication and engagement with friends and family outside of the project activities.

As such, controlling the parameters of the testing and intervention from a scientific perspective was compromised, and this was borne out by the quantitative results; the questionnaires proved challenging for some of the participants, which was not evident in initial piloting. However, changes and benefits did occur, and in terms of the social and emotional well-being of these particular older adults, this project has had a measurable impact.

With its myriad benefits in mind, this research demonstrates the value and impact of work of this nature, which creates an environment where the older adults feel both accepted by the other participants and connected to their respective networks beyond the project. The findings of this research suggest that continued support for projects like *Generations Dancing* will help to address the ongoing need to balance the health and well-being of both older adults and young people and in developing participants’ sense of community and civic safety by challenging assumptions and breaking down barriers for communication across generations.

## Data Availability Statement

The raw data supporting the conclusions of this article will be made available by the authors, without undue reservation, to any qualified researcher.

## Ethics Statement

The studies involving human participants were reviewed and approved by the University Ethics Committee, University of Bedfordshire. Written informed consent to participate in this study was provided by the participants’ legal guardian/next of kin.

## Author Contributions

LD wrote the manuscript. IA provided data for Tables 1, 2, plus contributed to the “Quantitative Methods” and “Quantitative Results” sections and conducted all statistical analyses. LD and RF conducted the participant interviews. All authors reviewed the final manuscript.

## Conflict of Interest

The authors declare that the research was conducted in the absence of any commercial or financial relationships that could be construed as a potential conflict of interest.
