# Supplementary material for: The Impact of an Intergenerational Dance Project on Older Adults’ Social and Emotional Well-Being
Source: Front Psychol. 2020 Sep 16;11:561126. doi: 10.3389/fpsyg.2020.561126 (PMC7525047; doi:10.3389/fpsyg.2020.561126)
Supplement: Supplementary file 1 [file Data_Sheet_1.PDF]

Appendix 1: Structure for focus groups

1.1 Older Adults

Have you been involved in any previous projects working in an arts/intergenerational context?

Why did you want to be part of this project?

    What were your expectations?

For you, how has it been?

    Process/final event?

What did you learn from it?

Did you feel that project addressed ideas of community and citizenship?

    If so how?

How has it been working with young people?

What's the experience been of collaborating between centres?

How was it when the groups were brought together?

Do you think there's value in this kind of project? Would you do it again?

Were there any challenges?

How did you find the structure/process of the project?

    How did you feel you contributed to the project?

    (How did you perceive the role of the university?)

    (In your opinion what were the pros and cons of working with an institution?)

Would you be interested in this developing into a longer-term/more regular project?

    How would it work for you?

    Is it a good thing to work up to a final showing?

Have you got anything else to add that we haven't covered?

1.2 Young People

Have you done anything like this before? Arts/intergenerational?

Why did you want to be part of this project?

What did you think it was going to be like and did it turn out that way?

For you, how has it been?

Process/final event?

What did you learn from it?

Does this link to anything you're doing at school?

Community/citizenship?

How has it been working with older people?

What's the experience been of working with the other school?

How was it when the older and younger groups were brought together?

Would you do it again? If so why – what did you get out of it?/ If not why?

Was there anything difficult about it or anything you didn't like?

How did you find the organisation of the project?

How did you feel you contributed to the project?

What was it like coming to the university?

(In your opinion what were the pros and cons of working with an institution?)

Would you be interested in this developing into a longer-term/more regular project?

How would it work for you?

Is it a good thing to work up to a final showing?

Have you got anything else to add that we haven't covered?

1.3 Artists

Can you tell me your date of birth?

Tell us about your background – how did you get into this kind of work

Have you had any previous experience working in an intergenerational context?

For you, how has it been?

Can you talk about the idea of community/citizenship in the work?

What sorts of things have you observed from the participants

> How engaged were they?
>
> Did they seem to enjoy it?
>
> Any differences between how the older and younger participants engaged?
>
> Did things change when you bought the groups together?
>
> Could you see any benefits when they were working together?
>
> Did they say anything to you about it?

Do you think there's value in this kind of project? Would you do it again?

What have been the challenges of the project?

How did you find the structure/process of the project?

> How did you see your roles in the project?
>
> How did you perceive the role of the university?
>
> In your opinion what were the pros and cons of working with an institution?

How could you see this develop into a longer-term/more regular project

> How would it work/would it work?
>
> Is it a good thing to work up to a final showing?

Have you got anything else to add that we haven't covered?

1.4 Scheme Managers

Can you tell me your date of birth?

Tell us about your background?

Could you tell us about your specific centre and the environment/ethos?

Why did you want to be part of this project?

  What were your expectations?

Have you been involved in any previous projects working in an arts/intergenerational context?

For you, how has it been?

  For you/the older adults?

How have you perceived the adults have engaged with the idea of community/citizenship in the work?

Does this link to any health/cultural/social initiatives?

What have your centres perception of it been – other staff/older adults?

What's the experience been of collaborating between centres?

What sorts of things have you observed from the older adults?

  How engaged were they?

  Did they seem to enjoy it?

  Did things change when the groups were brought together?

  Could you see any benefits when they were working together?

  Did they say anything to you about it?

  For the participants that dropped out why do you think that was?

Do you think there's value in this kind of project? Would you do it again?

What have been the challenges of the project?

How did you find the structure/process of the project?

  How did you see your roles in the project?

  How did you perceive the role of the university?

  In your opinion what were the pros and cons of working with an institution?

How could you see this develop into a longer-term/more regular project?

How would it work/would it work?

Is it a good thing to work up to a final showing?

Have you got anything else to add that we haven’t covered?

1.5 Teachers

Can you tell me your date of birth?

Tell us about your background?

Why did you want to be part of this project?

What were your expectations?

Have you been involved in any previous projects working in an intergenerational context?

For you, how has it been? For you/the students?

How have you perceived the students have engaged with the idea of community/citizenship?

Does this link to curriculum (SMSC) in terms of both citizenship and arts practice?

What have your schools perception of it been – other staff/students?

What's the experience been of collaborating between schools?

What sorts of things have you observed from the students?

How engaged were they?

Did they seem to enjoy it?

Did things change when the groups were brought together?

Could you see any benefits when they were working together?

Did they say anything to you about it?

For the participants that dropped out why do you think that was?

Do you think there's value in this kind of project? Would you do it again?

What have been the challenges of the project?

How did you find the structure/process of the project?

How did you see your roles in the project?

How did you perceive the role of the university?

In your opinion what were the pros and cons of working with an institution?

How could you see this develop into a longer-term/more regular project?

How would it work/would it work?

Is it a good thing to work up to a final showing?

Have you got anything else to add that we haven't covered?
