# Supplementary material for: The Impact of an Intergenerational Dance Project on Older Adults’ Social and Emotional Well-Being
Source: Front Psychol. 2020 Sep 16;11:561126. doi: 10.3389/fpsyg.2020.561126 (PMC7525047; doi:10.3389/fpsyg.2020.561126)
Supplement: Supplementary file 2 [file Data_Sheet_2.PDF]

Appendix 2: Statistical analysis results

Wilcoxon rank tests for change in variables among older adult experimental group. Please note that competence is missing from these results due to insufficient cases at Time 2.

	Z	***p***
**Autonomy**	-1.00	.32
**Relatedness**	-.37	.72
**Positive affect**	.00	1.00
**Negative affect**	-1.70	.09
**Social wellbeing**	-.27	.79

Paired sample t-tests for change in variables among young people experimental group

	t	**df**	***p***
**Autonomy**	-.50	1	.71
**Competence**	-.14	4	.90
**Relatedness**	-.82	6	.44
**Positive affect**	1.41	16	.18
**Negative affect**	1.31	15	.21
**Social wellbeing**	-.02	6	.98

Paired sample t-tests for change in variables among young people control group

	t	**df**	***p***
**Autonomy**	.33	2	.77
**Competence**	-5.28	4	.01
**Relatedness**	.68	3	.55
**Positive affect**	.12	10	.90
**Negative affect**	1.48	10	.17
**Social wellbeing**	-.18	10	.86

Independent t-tests for differences in scores between young people experimental and control groups at Time 1

	t	**df**	***p***
**Autonomy**	.30	20	.77
**Competence**	-.62	20.61	.55
**Relatedness**	1.05	20	.31
**Positive affect**	1.41	34	.47
**Negative affect**	.90	31.66	.38
**Social wellbeing**	-1.02	30	.32

Independent t-tests for differences in scores between young people experimental and control groups at Time 2

	T	**df**	***P***
**Autonomy**	-1.59	9.07	.15

**Competence**	-2.21	12.65	.05
**Relatedness**	.69	15	.50
**Positive affect**	.60	28	.55
**Negative affect**	.79	27	.44
**Social wellbeing**	-1.04	26	.31
